# Modulation of granulosa cell function via CRISPR-Cas fuelled editing of BMPR-IB gene in goats (*Capra hircus*)

**DOI:** 10.1038/s41598-020-77596-9

**Published:** 2020-11-24

**Authors:** Sai Kumar, Meeti Punetha, Bosco Jose, Jaya Bharati, Shivani Khanna, Arvind Sonwane, Jonathan A. Green, Kristin Whitworth, Mihir Sarkar

**Affiliations:** 1 Physiology and Climatology Division, ICAR-Indian Veterinary Research Institute, Izatnagar, Bareilly, Uttar Pradesh 243122 India; 2 Division of Animal Genetics, ICAR-Indian Veterinary Research Institute, Izatnagar, Bareilly, Uttar Pradesh 243122 India; 3 Division of Animal Science, University of Missouri-Columbia, Columbia, MO USA

**Keywords:** Biotechnology, Molecular biology, Physiology

## Abstract

BMPs are multifunctional growth factors implicated in regulating the ovarian function as key intra-ovarian factors. Biological effects of BMPs are mediated through binding with membrane bound receptors like BMPR-IB and initiating downstream Smad signaling pathway. FecB mutation, regarded as a loss of function mutation in the BMPR-IB gene was identified in certain sheep breeds having high fecundity. Similar type of fecundity genes in goats have not been discovered so far. Hence, the current study was designed to investigate the effects of BMPR-IB gene modulation on granulosa cell function in goats. The BMPR-IB gene was knocked out using CRISPR-Cas technology in granulosa cells and cultured in vitro with BMP-4 stimulation for three different durations In addition, the FecB mutation was introduced in the BMPR-IB gene applying Easi-CRISPR followed by BMP-4/7 stimulation for 72 h. Steroidogenesis and cell viability were studied to explore the granulosa cell function on BMPR-IB gene modulation. BMPRs were found to be expressed stage specifically in granulosa cells of goats. Higher transcriptional abundance of R-Smads, LHR and FSHR indicating sensitisation of Smad signaling and increased gonadotropin sensitivity along with a significant reduction in the cell proliferation and viability was observed in granulosa cells upon BMPR-IB modulation. The inhibitory action of BMP-4/7 on P4 secretion was abolished in both KO and KI cells. Altogether, the study has revealed an altered Smad signaling, steroidogenesis and cell viability upon modulation of BMPR-IB gene in granulosa cells similar to that are documented in sheep breeds carrying the FecB mutation.

## Introduction

Cyclical production of fertilizable ova and steroid hormones are the two major functions performed by mammalian ovary^1^. The ovarian follicle, regarded as a fundamental unit of the mammalian ovary endows the necessary microenvironment for oocyte growth, maturation and performs some complex reactions that yield important steroid hormones^2,3^. Follicular growth can be divided gonadotropin independent, gonadotropin responsive and gonadotropin dependent phases depending on their gonadotropin dependence^4–6^. In the gonadotropin responsive phase, growth of the follicle does not strictly require gonadotropins for growth but stimulated if present and chiefly regulated by intraovarian regulators like growth factors, cytokines, and gonadal steroids^7^. The emergence of the dominant follicle in each follicular wave depends on the tissue specific expression of the BMP system that includes ligands and their receptors^8^.

BMPs, regarded as indispensable group of multifunctional growth factors belonging to the TGF-β superfamily^9^. Apart from possessing the distinct ability to induce bone, cartilage, ligament, and tendon formation, BMPs also play a critical role in the regulation of cell proliferation, survival, differentiation and apoptosis. BMPs bind to a hetero-tetrameric transmembrane receptor complex composed by type I (BMPR-IA and BMPR-IB) and type II (BMPR-II) serine threonine kinase receptors^10^. The signal transduction of BMPs via their receptors occurs via Smad dependent or independent pathways, the former being the customary pathway^11^. The canonical Smad dependent pathway recruit Smads as major signal transducers for the serine/threonine kinase receptors in BMP signaling. Activation of type I receptors by ligand bound type-II receptors leads to the phosphorylation of receptor-regulated Smads (R-Smads), resulting in the formation of complexes with common-partner Smads (Co-Smads). Translocation of R-Smad/Co-Smad complexes into the nucleus helps in regulating transcription of target genes by interacting with various transcription factors and transcriptional co-activators or co-repressors^10,12^. Point mutation in the BMPR-IB gene resulting in an A → G substitution at 746 position in exon eight is known as the FecB or Booroola mutation, leading to the substitution of the 249th amino acid from glutamine to arginine (Q249R) known as FecB or Booroola fecundity gene^13^. Regarded as a loss of function mutation, FecB found to exert profound effect on litter size and ovulation rate in Booroola Merino sheep^14,15^. Each copy of mutant allele increases ovulation rate by about 1.6 with the effect being additive for an additional copy^16^. Introgression of FecB gene into non-prolific sheep breeds like Malpura and Kashmir valley lead to a significant improvement in their fecundity^17,18^. FecB mutation induces precocious maturation of follicles, increased responsiveness to FSH and progesterone production^8^. The presence of large number of small pre-ovulatory follicles is one of the striking features identified in FecB carrier ewes^19^. However, the FecB mutation was found to be absent in Indian goat breeds viz. Black Bengal, Beetal, Barbari, Malabari, Sikkim, Jakhrana, Raighar and Gaddi^20,21^. Goat, known as a “poor man’s cow”/“mini cow”, holds a prime position in providing livelihood activity and subsidiary income to many landless and marginal farmers in India. Recent reports suggest a negative growth trend with an estimated 3.82 per cent decline in goat population over the previous census (19th Livestock Census, 2012). Augmenting the reproductive efficiency of low prolific breeds is need of the hour to counter the dwindling numbers and amass goat population that leads to a subsequent increase in Chevon production.

Site-specific genetic engineering has become effortless with the advent of Clustered Regularly Interspaced Short Palindromic Repeats (CRISPR-Cas) genome editing technology and an indispensable tool for functional validation of genes. Nevertheless, the genetic control for fecundity in goats remains as a key area to be addressed for the aforementioned reasons. However, we hypothesised that modulation of BMPR-IB gene may alter the granulosa cell function in goat similar to what was found in sheep breeds carrying the FecB mutation. Hence, the current study was designed to investigate the effects of BMPR-IB gene modulation on granulosa cell function in terms of steroidogenesis, gonadotropin sensitivity and cell survivability.

## Methods

All methods and experimental protocols were carried out in accordance with relevant safety guidelines and regulations.

### Experiment 1

To demonstrate the transcriptional abundance of BMPRs in granulosa cells in follicles during different stages of growth in goats.

### Isolation of granulosa cells (GC)

Ovaries from apparently healthy does without any abnormalities in the reproductive tract were collected from the local slaughterhouse and transported to the laboratory at 37 °C in sterile PBS supplemented with antibiotic- antimycotic solution (Penicillin, Streptomycin and Amphotericin-B). Up on gross examination of the follicles contour and vascularity, healthy follicles were chosen and classified in to three categories based upon their diameter as Small (< 3 mm), Medium (3-5 mm) and large (> 5 mm)^22–24^. Granulosa cells from each follicle class (n = 6) were harvested according to the earlier described protocol^25^.

### RNA extraction and cDNA synthesis

Total RNA was extracted from different GC samples using Trizin reagent (GCC Biotech) according to the manufacturer’s protocol. Followed by RNA extraction, the RNA samples were subjected to RNase free DNase treatment with subsequent incubation at 56 °C for 10 min to inactivate DNAase. The quality and integrity of the RNA samples was checked by A260/A280 ratio using a Nanodrop spectrophotometer along with visualising 28S and 18S bands using agarose gel electrophoresis. 0.5 µg of total RNA was reverse transcribed to yield cDNA using RevertAid First cDNA synthesis kit (ThermoFisher Scientific).

### Primers

Primers for BMPR-IA, BMPR-IB and RPS15A were designed in-silico using the DNAStar (online trial version, DNASTAR Lasergene 6, 2004), Gene tool (online trial version, 2004) and Oligo Analyser (open access tool, 2017) software. Previously published primer was used for BMPR-II. Details of the primers are listed in Table 1. Annealing temperature of each primer was optimised using end point PCR.

**Table 1:** Primer sequences used for target genes in the current study.

Gene	Sequences of nucleotide (5′–3′)	Amplicon length (bp)	EMBL accession no.(or) reference
BMPR-IA	**Forward: **GTGTCACAGGAGGAATAGTCGAAG **Reverse:** GACACAATTGGCCGCAAACG	121	XM_018042146.1
BMPR-IB	**Forward:** TGTGTGTCAGGAGGTATAGTGG **Reverse:** TGAGACACTCGTCACTGCTC	151	XM_013964506.2
BMPR-II	**Forward:**TGTGCCAAAGATTGGCCCTT **Reverse:** TGCTTGCTGCCGTTCATAGT	175	J. J. N. Costa et al., 2012
RPS-15A	**Forward:**AGGGCTGGGAAAATTGTTGTGAA **Reverse:**TGACGGGATGGGAGCAGGTTAT	125	XM_005697526.3
SMAD-1	**Forward:** TCTTTCCAGCAGCCCAACAGC **Reverse:** CTGGTCGGAGAGTGAGGGTA	104	XM_018061578.1
SMAD-5	**Forward:** CCTATGAAGAGCCCAAACAT **Reverse:** GGTCTGTGAATCCATCTACTA	114	XM_018050347.1
SMAD-8	**Forward:** ACTATCAGCACGGCTTCCAC **Reverse:** GGTCAGCTCATAGACGACTTC	140	XM_005687505.3
StAR	**Forward:** CTGCGTGGATTAACCAGGTTCG **Reverse:** CAAGCTCTTGGTCGCTGTAGAG	84	XM_013975437.2
3βHSD	**Forward:** CGGCATCCTGACCAATTACT **Reverse:** TTTGGTGTGGTGTGTCGTCT	164	50
CYP11A1	**Forward:** ATCCACTTTCGCCACATCG **Reverse:** GGTCTTTCTTCCAGGCTCCT	232	51
CYP19A1	**Forward:** CATCCTCAATACCAGGTCCCA **Reverse:** GGTTTCCTCTCCACATACCCA	152	51
PCNA	**Forward:** TACCTGTAGCCGTGTCATTGCGA **Reverse:** AGACGAGTCCATGCTCTGTAGG	162	XM_005688167.3
CASPASE3	**Forward:** TTCCTGGCGAAACTCAAAGT **Reverse:** TTGCATGAAAAGCAGAATCG	164	XM_018041755.1
LHR	**Forward:** GAAAGCACAGCAAGGAGACC **Reverse:** CAGTCACATTTCCCGTGATG	226	NM_001314279.1
FSHR	**Forward:** AGGCAAATGTGTTCTCCAACCTGC **Reverse:** TGGAAGGCATCAGGGTCGATGTAT	91	52
SgRNA	**Forward:** TAATACGACTCACTATAGAGATTGGAAAAGGTCGCTAT **Reverse:** TTCTAGCTCTAAAACATAGCGACCTTTTCCAATCT		
Genomic cleavage detection	**Forward:** TCTGTAGTGCCGTGAATGCA **Reverse:** CATTCTTGGGCTCATGGCAA		
ssODN	CTACAGAGGAGGCCAGCTGGTTC**CGAGAGACAGAAATATATAGGACGGTGTTG**ATGAGGCATGAAAACATCTT		

### qPCR analysis

Quantitative real-time PCR was performed using the Maxima SYBR Green qPCR kit (Thermo Scientific). The slopes obtained by the amplification of a standardized dilution series were used for determining Real time PCR efficiencies. A generalised qPCR protocol was followed with initial denaturation at 95 °C for 10 min followed by 40 cycles of denaturation at 95 °C for 15 s, with primer specific annealing temperature step and extension at 72 °C for 30 s. A no template control reaction was set up for each primer to monitor the contamination and primer-dimer formation. The cycle threshold values (C_t_), amplification plot and melt curves were analysed using the Biorad CFX manager Real-Time qPCR software.

#### Experiment 2

To demonstrate the effect of knocking out of BMPR-IB gene on granulosa cell function in goats.

### Tissue collection and granulosa cell (GC) culture

**Figure 1:**
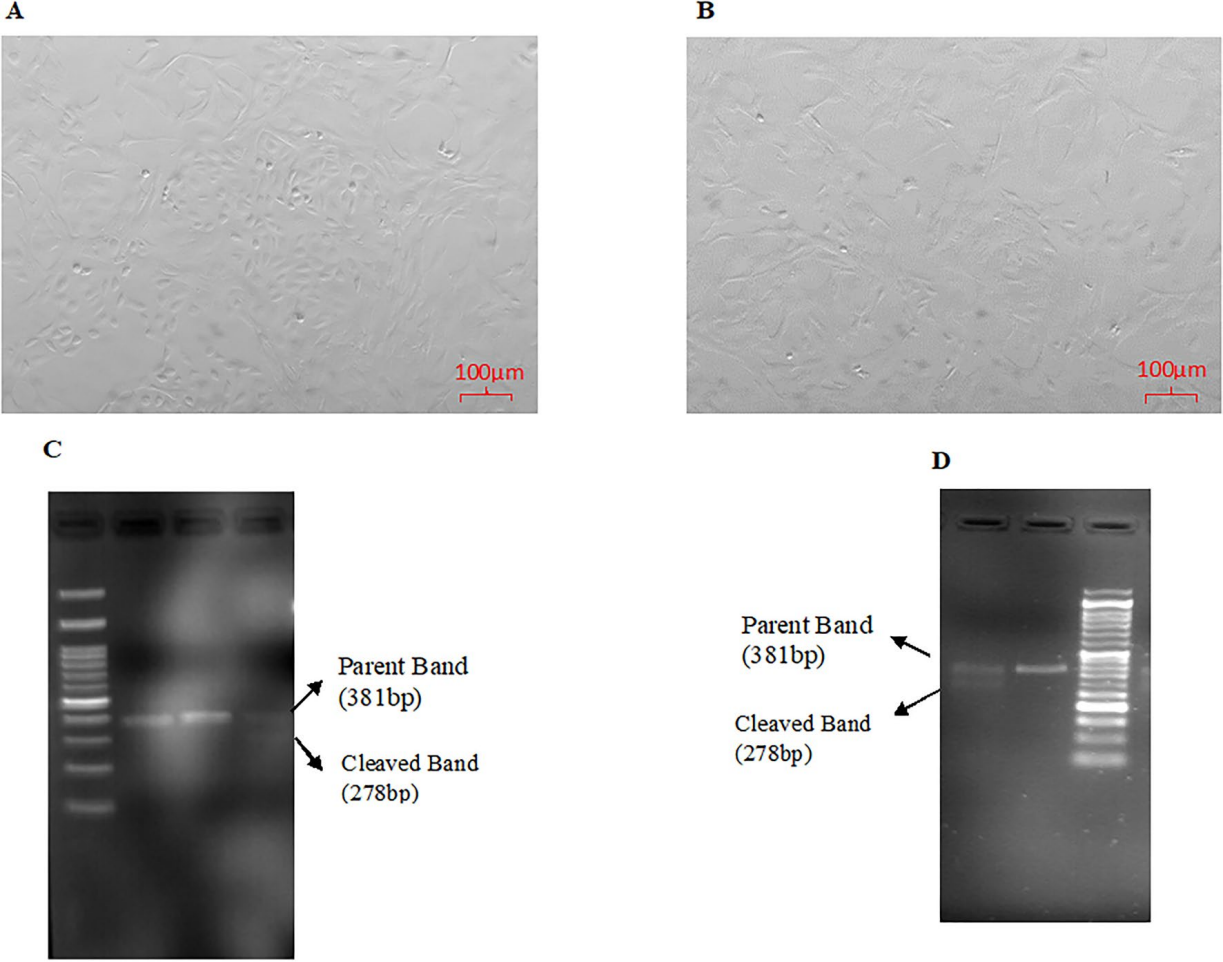
(**A**) 75–80% in vitro cultured caprine granulosa cells. (**B**) In vitro cultured BMPR-IB KO cells. (**C**) Gel image of Genomic Cleavage Detection Assay of granulosa cells transfected with components of CRISPR-Cas system for BMPR-IB gene knock out (KO) using Lipofectamine 2000. Lane 1 and 2: Negative control samples for BMPR-IB KO, Lane3: Genomic cleavage showing both Parent band and Cleaved band. (**D**) Gel image of Genomic Cleavage Detection Assay of granulosa cells electroporated with components of CRISPR-Cas system along with ssODN for BMPR-IB gene knock-in (KI) using Lipofectamine 2000. Lane1: Genomic cleavage showing both Parent band and Cleaved band and Lane2: Negative control sample for BMPR-IB knock-in (KI).

A primary granulosa cell culture from large antral follicles (> 5 mm in diameter) according to the established protocol^26^. Briefly, the follicular fluid (FF) was aspirated using a sterile needle (22 gauge) and syringe. Then, the contents were transferred into a Petri plate (60 mm) containing 1X PBS followed by the removal of all cumulus-oocyte complexes (COCs). The remaining cells in the PBS were transferred into a 15 mL centrifuge tube followed by centrifugation at 300 g for 5 min. The supernatant was discarded and the GC pellet obtained was again subjected to a second wash with 1X PBS and centrifuged. The GC pellet was then resuspended in Dulbecco’s modified Eagle’s medium (DMEM) (Hyclone) containing 10% fetal bovine serum (FBS) and antibiotic–antimycotic solution. Trypan blue exclusion dye technique was used to assess the vitality of the cells. Finally, then the granulosa cells were seeded at a density of 2 × 10^5^ viable cells/well in a 24-well plate and cultured in a humidified CO_2_ (5%) incubator at 37 °C (Fig. 1A).

### Production of BMPR-IB knock out (KO) granulosa cells (GC)

Knocking out of BMPR-IB gene in granulosa cells (Fig. 1B) was accomplished by using CRISPR-Cas genome editing technology. The CRISPR-Cas machinery was delivered into the cells using lipofection and the workflow was described below:

### Preparation of synthetic single guide RNA (SgRNA)

SgRNA for targeting the BMPR-IB gene was designed in silico using available software. The synthesis of SgRNA was performed according to the laboratory protocol established earlier^27,28^. Prior to the SgRNA synthesis, T7 promotor sequence was added to the forward primer and the DNA template for SgRNA was generated by PCR amplification using the designed primers. Then, the SgRNA was obtained by performing in vitro transcription (IVT) using High TranscriptAid Enzyme Mix (Invitrogen) and later the in vitro transcribed SgRNA was purified using the GeneJET purification. The quality of the SgRNA was assessed by agarose gel electrophoresis and quantified using Nanodrop spectrophotometry.

### Transfection and genomic cleavage detection

Ready to transfect Cas9 was procured from (GeneArt Platinum Cas9 Nuclease Cat. No. B25640). The SgRNA and Cas9 (RNP) were added to the cultured granulosa cells (75–80% confluency) using Lipofectamine as per manufacturer’s protocol (Invitrogen; Cat. No. CMAX00015). The transfected cells were cultured for 72 h and T7E1 assay (GeneArt Genomic Cleavage Detection Kit, Invitogen; Cat No. A24372) was used to assess the cleavage efficiency (Fig. 1C).

The cleavage efficiency of the BMPR-IB knock out was measured by the following equation:

$$ \text{Cleavage efficiency} = [\text{sum of cleaved band intensities}]/(\text{sum of cleaved and parental band intensities})\times 100\%^{29}. $$

### Treatment of BMPR-IB KO cells with BMP-4

The wild type cells and KO cells were then cultured for three different durations (24, 48 and 72 h) with BMP-4 @50 ng/mL^30^. Altogether, there were four (4) experimental conditions: (1) Wild type cells, (2) Wild type cells treated with BMP-4, (3) KO cells and (4) KO cells treated with BMP-4. Cells were cultured in replicates of four in each group. Followed by the completion of each duration, MTT assay was performed to determine the cell viability, the spent media was stored at − 20 °C for estimating progesterone (P4) and estradiol (E2) concentrations and cells were harvested for total RNA isolation. Finally, qPCR analysis of StAR, 3βHSD, CYP11A1 (P450scc), CYP19A1 (Aromatase), Smad-1, Smad-5, Smad-8, Caspase3, PCNA, LHR and FSHR was carried out to determine the expression pattern.

#### Experiment 3

To investigate the effects of introduction of Booroola (FecB) mutation [knock-in] on granulosa cell function in goats.

### Production of BMPR-IB Knock-in (KI) granulosa cells and detection

The FecB mutation in BMPR-IB gene was introduced using Easi-CRISPR^31^. The SgRNA used earlier in the knock out experiment along with the ssODN template having the desired point mutation were used for knocking-in.

### Preparation of single-stranded oligo deoxynucleotides (ssODN) containing Booroola (FecB) mutation

Single-stranded oligo deoxynucleotides (ssODN) containing Booroola (FecB) mutation (~ A746G) was designed and used as a HDR template. The ssODN with the desired mutation was flanked by two homologous arms (arm length 30–60 bases) on each side. An additional synonymous mutation (C → A), 1 bp upstream of the target site, was designed in the synthetic ssODN to identify the desired change by genomic cleavage detection assay.

### Transfection and genomic cleavage detection

The Cas9, SgRNA and ssODN were delivered using Neon transfection system (Invitrogen). Prior to the delivery, the electroporation conditions were optimised (Voltage: 1200 V, Width: 20 ms, Frequency: 2) in granulosa cells using plasmid encoded with green fluorescent (GFP). After electroporation, the cells were seeded in a 24 well culture plate and cultured for a period of 72 h. The knock-in was detected using the T7E1 assay (Fig. 1D) (GeneArt Genomic Cleavage Detection Kit, Invitrogen) as described in KO.

### Treatment of BMPR-IB KI cells with BMP-4 and BMP-7

The wild type and knock-in cells were then treated with BMP-4 (50 ng/ml)and BMP-7 (100 ng/ml) separately for 72 h. In each treatment there were 4 experimental conditions: (1) Wild type cells, (2) Wild cell with BMP-4 /BMP-7, (3) Knock-in and (4) Knock-in cells with BMP-4/BMP-7. After 72 h, cell viability was assessed by MTT assay, spent media was stored for P4 and E2 estimation and the cells were harvested for total RNA extraction. The total RNA extracted was used for expression analysis of StAR, 3βHSD, CYP11A1 (P450scc), CYP19A1 (Aromatase), Smad-1, Smad-5, Smad-8, Caspase3, PCNA, LHR and FSHR genes.

### Cell viability assay

MTT assay was performed to assess the cell viability in both the experiments. Briefly, the cells were seeded on 96 well plates and cultured at 37 °C and were incubated for prescribed durations. After culture, 10 μl of 5 mg/ml of 3-(4,5-dimethylthiazole-2-yl)-2,5-diphenyltertrazolium bromide (MTT; MP Biomedicals) was added in to each well to get a final concentration of 0.5 mg/mL media/well. The cells were further incubated for 4 h at 37 °C and followed by discarding the media along with MTT reagent. Then, 100 μl of DMSO (MP Biomedicals) was added and the absorbance at 450 nm recorded (Biorad, Microplate reader) within 15 min.

### Hormone estimation

Concentrations of P4 in the spent media was determined by P4 125I RIA kit (Immunotech, Czech Republic) as per manufacturer's instructions. Concentration of E2 was determined in the spent media using E2 ELISA kit (LDN).

### Statistical analyses

The resultant experimental data are shown as Mean ± SEM. Statistical significance differences in expression analysis of different genes chosen in the current study, P4 and E2 concentrations and cell viability were assessed using the software SPSS.22 (online trial version) by using one-way ANOVA followed by Tukey’s honestly significant difference (HSD) test. The relative quantification of mRNA was obtained using Pfaffl’s method^32^. Differences were considered significant at *p* < 0.05.

## Results

### Transcriptional abundance of BMPRs in caprine granulosa cells

**Figure 2:**
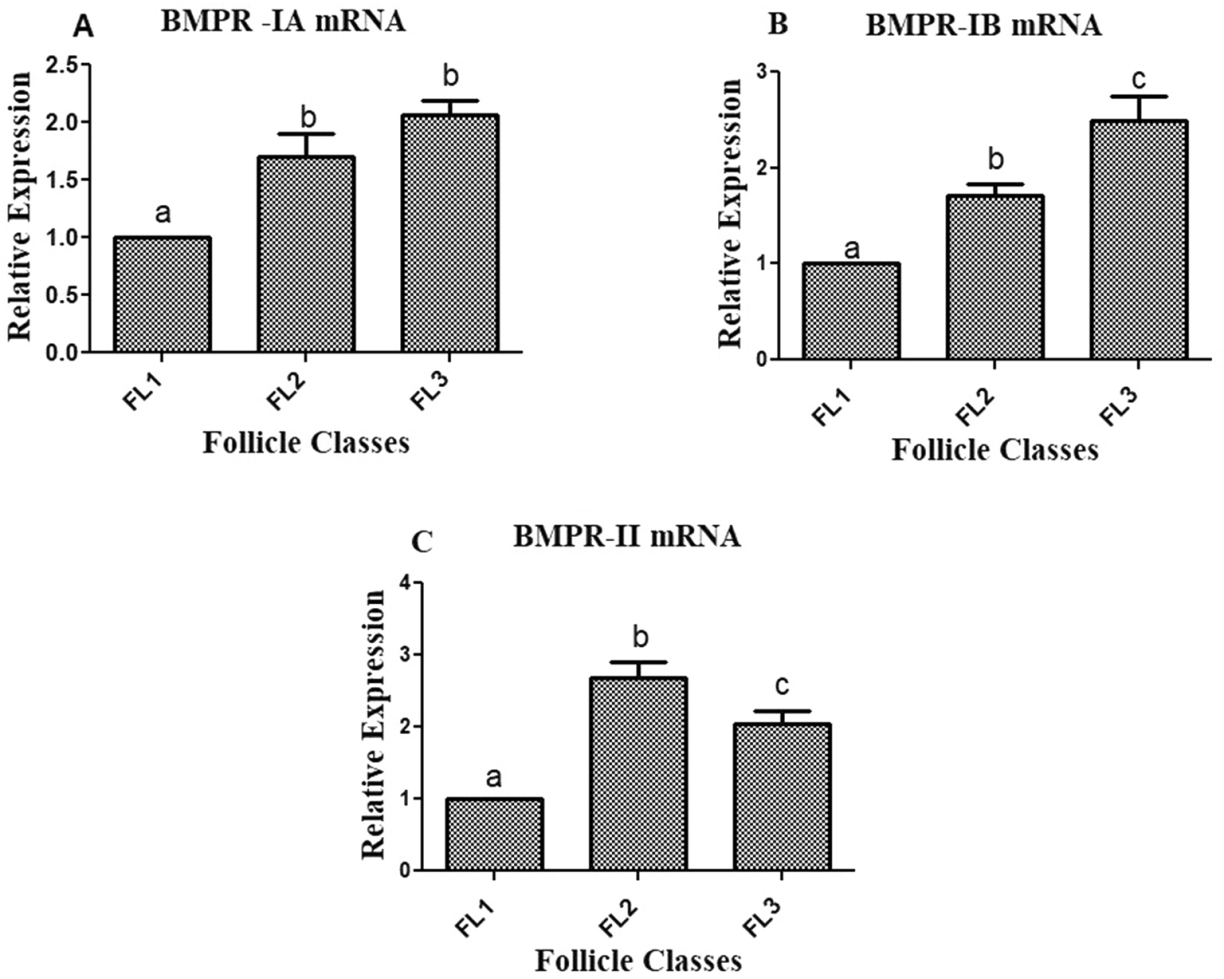
Transcriptional abundance of BMPRs (BMPR-IA, BMPR-IB and BMPR-II) in granulosa cells from different antral follicles of Goat (n = 6/group). All values are shown as mean ± SEM. Different superscripts denote statistically different values (*p* < 0.05). Abbreviations: FL1, FL2 and FL3 denotes antral follicles of < 3 mm, 3–5 mm and > 5 mm diameters respectively.

Transcripts of BMPR-IA were significantly upregulated in medium and large antral follicles as compared to the small antral follicle (*p* < 0.05; Fig. 2A). Whereas, the relative fold change of BMPR-IB mRNA was significantly higher in large follicles, followed by medium follicles (*p* < 0.05; Fig. 2B). BMPR-II was significantly upregulated **i**n the medium stage follicle and a significant downregulation in the large antral follicles was observed (*p* < 0.05; Fig. 2C). Overall, all the three variants of BMP receptor system were being expressed in the granulosa cells of goats.

### Effects of BMPR-IB gene KO on granulosa cells

#### Effects on Smad signaling

**Figure 3:**
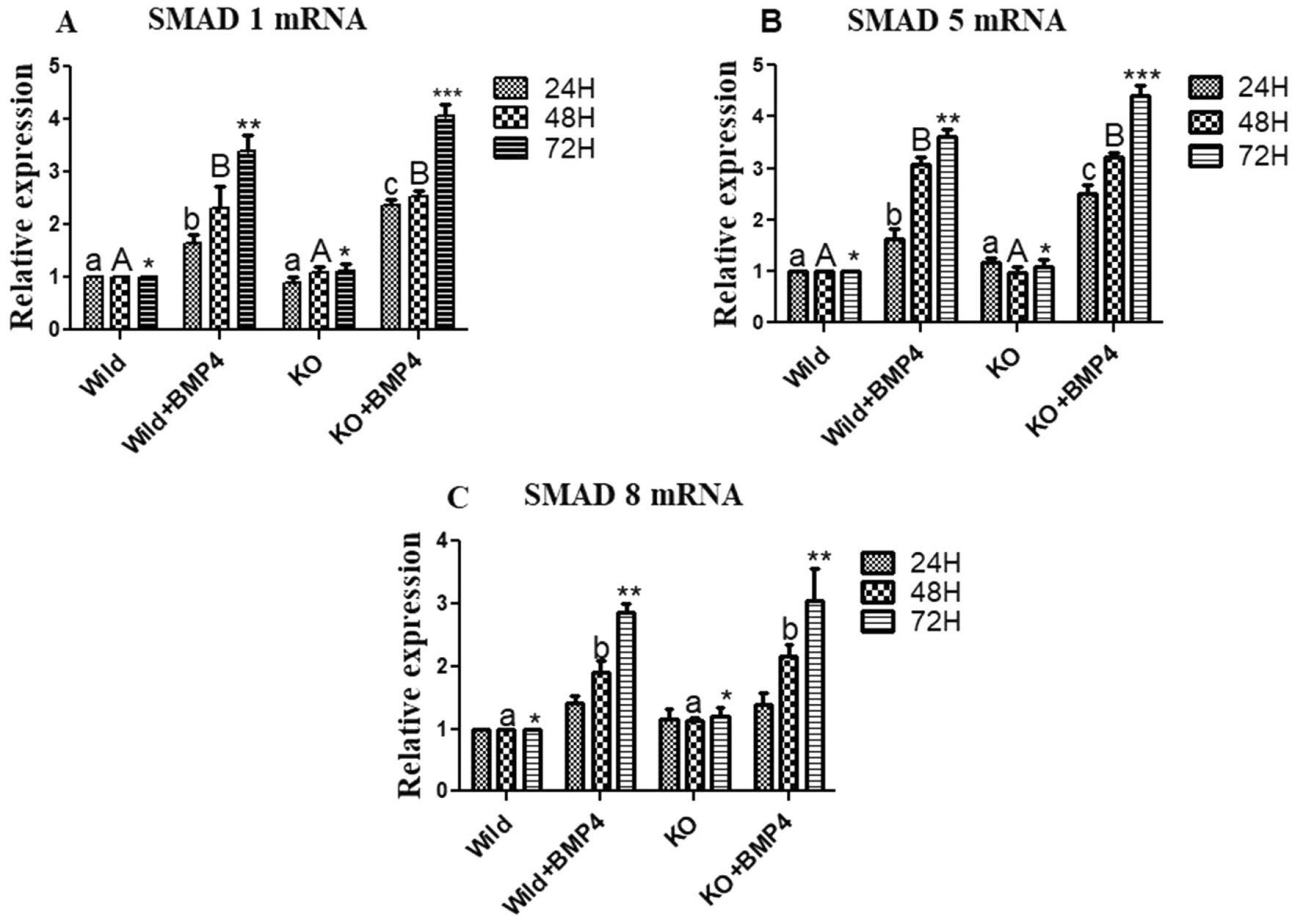
Effect of BMPR-IB gene KO on expression of Smad1, Smad5 and Smad 8 transcripts in in vitro cultured granulosa cells stimulated with BMP-4 (50 ng/ml) for three different time durations (24, 48 and 72 h). All values are shown as mean ± SEM. Different superscripts denote statistically different values (*p* < 0.05). Abbreviations: WT indicate wild type cells, WT + BMP-4 indicate wild cells treated with BMP-4 (50 ng/mL), KO indicate BMPR-IB gene knock out cells. KO + BMP-4 indicate KO cells treated with BMP-4(50 ng/ml).

Smad-1 expression was significantly up regulated (*p* < 0.05; Fig. 3A) in both the wild and KO cells treated with the BMP ligand in all the three durations, with the highest expression being in the latter. Expression analysis of Smad-5 revealed a significant up regulation (*p* < 0.05; Fig. 3B) of Smad-5 in all the three durations in both wild and KO cells cultured with BMP-4 in comparison to their control groups. However, the expression was higher in KO cells with BMP-4 than wild cells cultured with BMP-4. On the other hand, no significant difference in the expression of Smad-8/9 was found in granulosa cells cultured for 24 h. Nevertheless, the levels are significantly up regulated from 48 h onwards in both the WT and KO cells cultured with BMP-4(*p* < 0.05; Fig. 3C). Taken together, there is an increased expression of R-Smads in BMPR-IB gene knock out cells cultured with BMP-4 in comparison to the wild cells treated with BMP-4 and the control groups.

#### Effects on granulosa cell steroidogenic pathway and gonadotropin sensitivity

**Figure 4:**
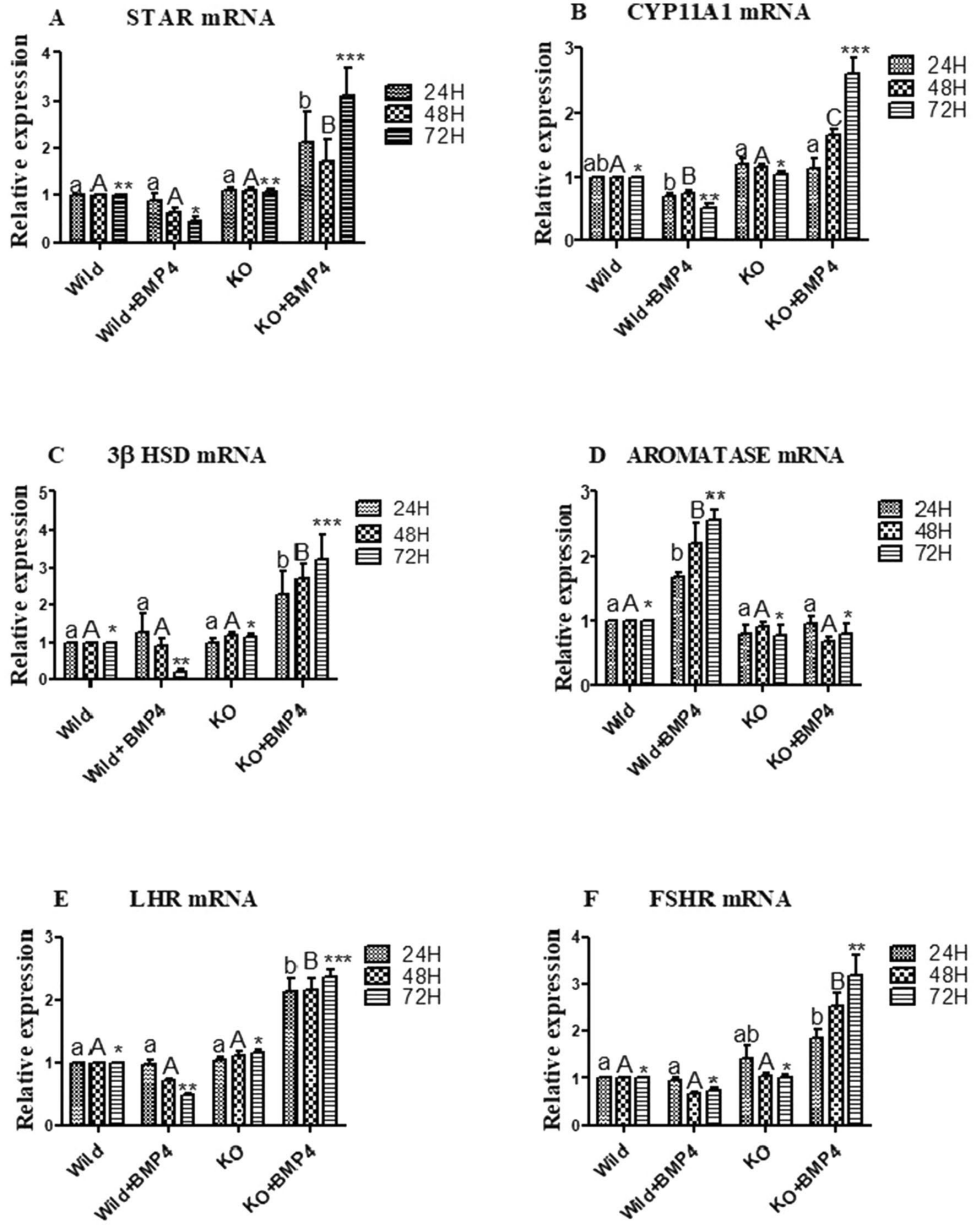
Effect of BMPR-IB gene KO on expression pattern of StAR, CYP11A1, 3βHSD, Aromatase, LHR and FSHR genes in in vitro cultured granulosa cells stimulated with BMP-4 (50 ng/ml) for three different time durations (24, 48 and 72 h). All values are shown as mean ± SEM. Different superscripts denote statistically different values (*p* < 0.05). Abbreviations: WT indicate wild type cells, WT + BMP-4 indicate wild cells treated with BMP-4 (50 ng/mL), KO indicate BMPR-IB gene knock out cells. KO + BMP-4 indicate KO cells treated with BMP-4(50 ng/ml).

The expression of StAR transcripts in the WT cells treated BMP-4 showed a decreasing trend with a significant fall in the 72 h group. On the other side, the expression in KO cells stimulated with BMP-4 increased significantly in comparison to all other groups (*p* < 0.05; Fig. 4A). The mRNA expression of CYP11A1 gene followed the same trend as that of StAR, with a significant fall in the WT cells stimulated with BMP-4 (at 48 and 72 h) and up regulated in all the three durations in KO cells cultured with BMP-4 (*p* < 0.05; Fig. 4B). Expression analysis of 3βHSD transcripts revealed a significant increase in the KO cells cultured with BMP-4 in all the three durations, while a decreasing trend, which was significant at 72 h, has been observed in the wild cells stimulated with BMP-4(*p* < 0.05; Fig. 4C). The aromatase (CYP19A1) expression in WT cells was on an average 3 folds higher in and significantly differ from the other groups including the KO cells supplemented with BMP-4 (*p* < 0.05; Fig. 4D). Expression analysis of LHR revealed a significant increase in BMP-4 stimulated KO cells, a significant down regulation was seen in WT cells treated with BMP-4 at 72 h (*p* < 0.05; Fig. 4E). FSHR transcripts in BMP-4 treated WT cells were on a decreasing trend, which is insignificant. On the other side, FSHR transcripts in KO cells treated with BMP-4 were significantly upregulated at all the three durations (*p* < 0.05; Fig. 4F).

#### Effects on cell proliferation and viability

**Figure 5:**
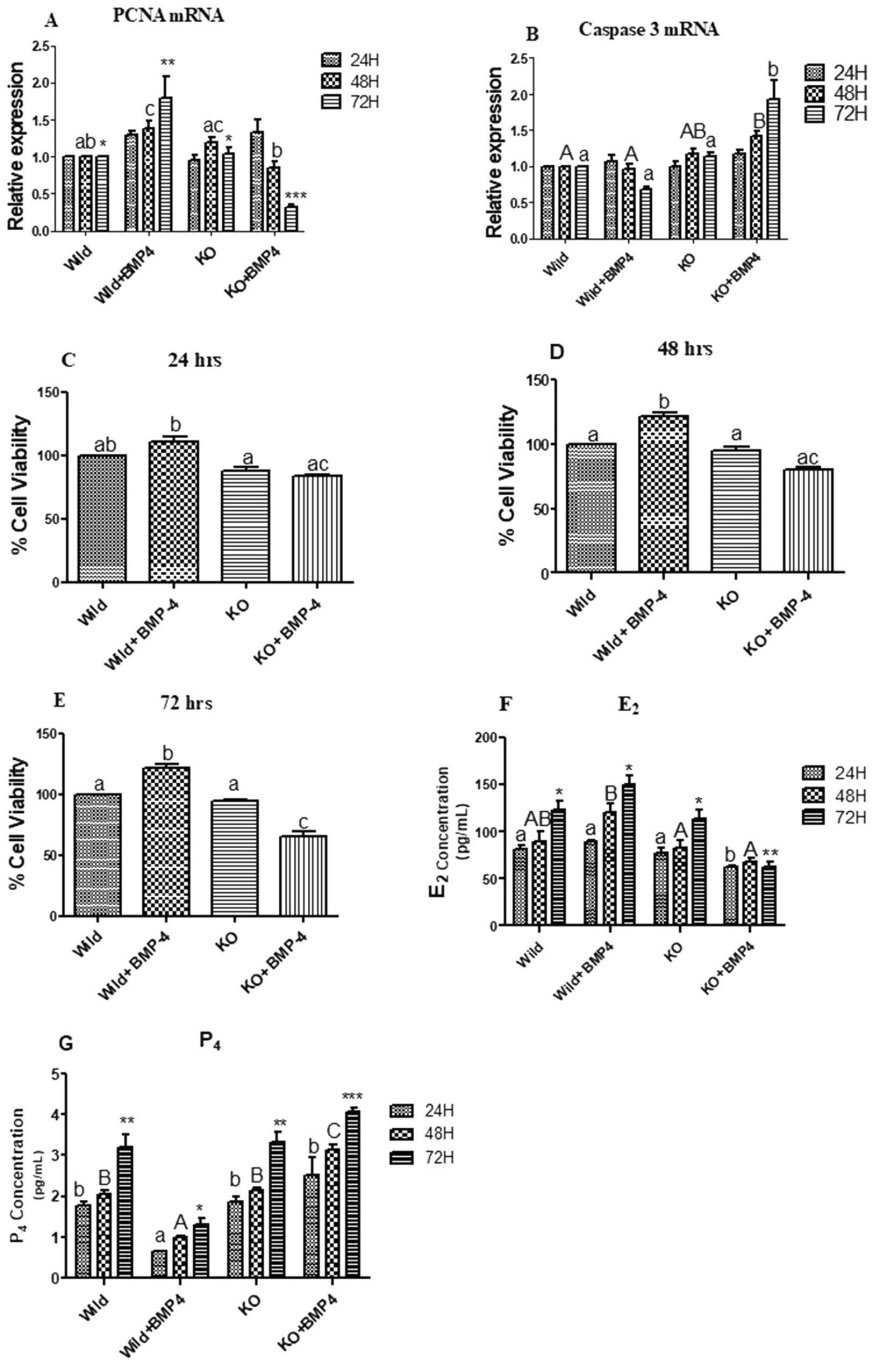
(**A**, **B**) Effect of BMPR-IB gene KO on expression pattern of PCNA, Caspase3 genes on in vitro cultured granulosa cells stimulated with BMP-4 (50 ng/ml) for three different time durations (24, 48 and 72 h). All values are shown as mean ± SEM. Different superscripts denote statistically different values (*p* < 0.05). Abbreviations: WT indicate wild type cells, WT + BMP-4 indicate wild cells treated with BMP-4 (50 ng/mL), KO indicate BMPR-IB gene knock out cells. KO + BMP-4 indicate KO cells treated with BMP-4(50 ng/ml). (**C**, **D**, **E**) Effect of BMPR-IB gene KO on cell viability in in vitro cultured granulosa cells stimulated with BMP-4 (50 ng/ml) for three different time durations (24, 48 and 72 h) assessed by MTT assay. All values are shown as mean ± SEM. Different superscripts denote statistically different values (*p* < 0.05). Abbreviations: WT indicate wild type cells, WT + BMP-4 indicate wild cells treated with BMP-4 (50 ng/mL), KO indicate BMPR-IB gene knock out cells. KO + BMP-4 indicate KO cells treated with BMP-4(50 ng/ml). (**F**, **G**) Effect of BMPR-IB gene KO on Progesterone (P4) and Estradiol (E2) stimulation of BMPR-IB gene KO on All values are shown as mean ± SEM. Different superscripts denote statistically different values (*p* < 0.05). Abbreviations: WT indicate wild type cells, WT + BMP-4 indicate wild cells treated with BMP-4 (50 ng/mL), KO indicate BMPR-IB gene knock out cells. KO + BMP-4 indicate KO cells treated with BMP-4(50 ng/ml).

Significant up regulation of PCNA in wild cells supplemented with BMP-4 at 48 and 72 h in comparison to the control group (wild cells without treatment) was observed. On the other side, there is a decreasing trend in PCNA expression in KO cells with BMP-4 significantly differing BMP-4 treated wild cells at 48 and 72 h (*p* < 0.05; Fig. 5A). With an overall increasing trend, a significant up regulation was observed in the Caspase3 expression at 48 and 72 h in knock out cells with BMP-4 when compared with wild cells with or without BMP-4 treatment (*p* < 0.05; Fig. 5B). MTT assay in KO cells stimulated BMP-4 revealed a significant decrease in the number of viable cells over a period of 24, 48 and 72 h (*p* < 0.05; Fig. 5C,D,E). On the other side, there is a significant increase at 48 and 72 h period in the viable WT cell numbers with BMP-4 treatment. Furthermore, the cell viability results were in accordance with the expression pattern of PCNA and Caspase3 genes.

#### Effect on estradiol (E2) and progesterone (P4) production

Significantly higher E2 concentrations were observed at 48 and 72 h in the WT cells treated with BMP-4 in comparison to KO cells treated with BMP-4 (*p* < 0.05; Fig. 5F). On the other side, the E2 concentration in BMP-4 treated KO cells was on the lower end when compared to the untreated knock out and wild cells. These observed results were in agreement with the lowered and increase expression of aromatase in treated KO and wild cells respectively. P4 concentrations in the WT cells treated with BMP-4 was significantly lower in comparison to the untreated wild cells, untreated KO cells and KO cells treated with BMP-4 at all the durations. The P4 concentrations were on the higher in BMP-4 treated KO cells with a significant difference at 48 and 72 h when compared to the rest of the groups (*p* < 0.05; Fig. 5G). These results are in congruence with the altered expression of StAR, CYP11A1 (P450scc), 3βHSD and CYP19A1 found in wild and KO cells treated with BMP-4.

#### Effects of the introduction of FecB mutation on granulosa cell function

##### Effects on Smad signaling

**Figure 6:**
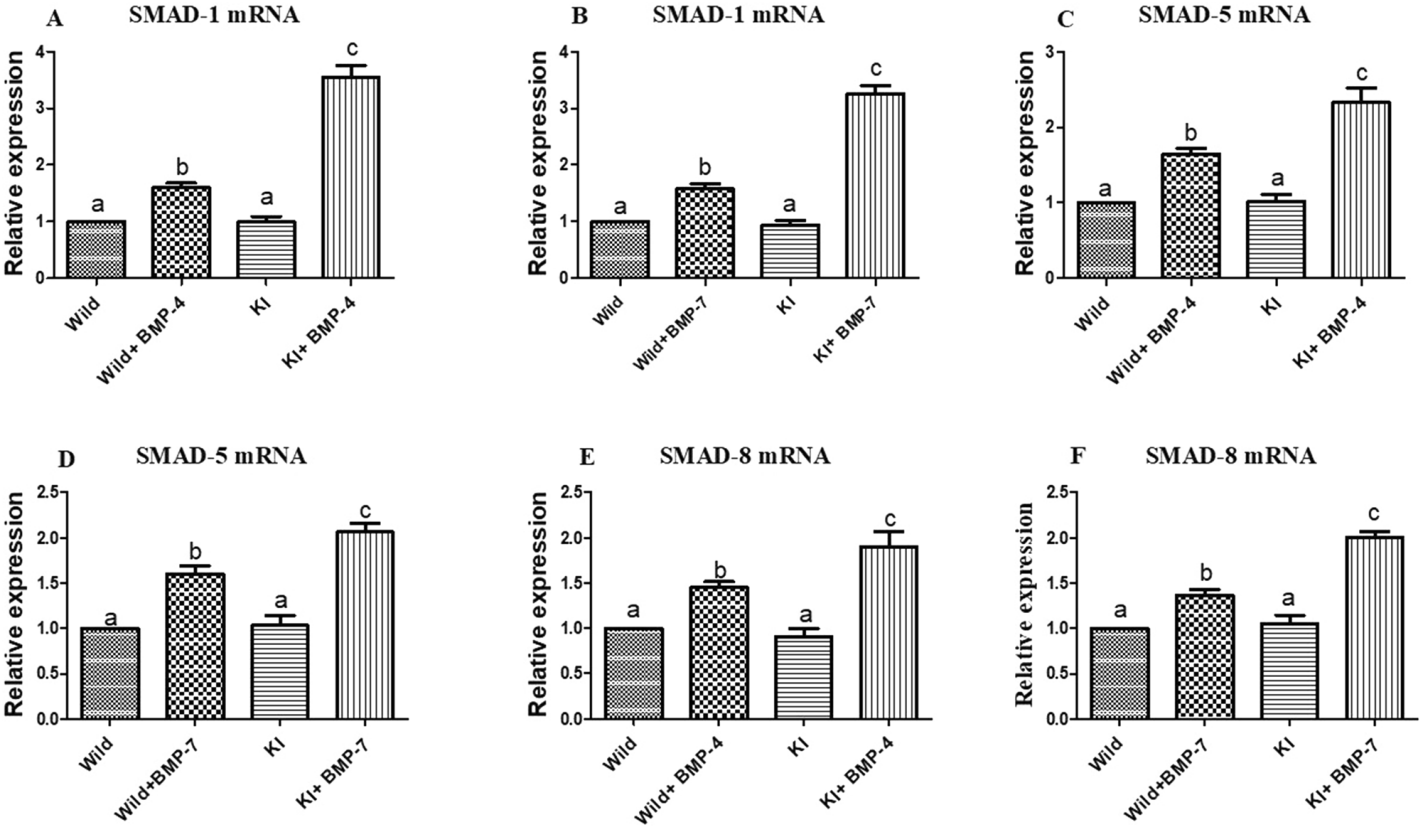
Effect of BMPR-IB gene KI on expression of Smad1, Smad5 and Smad 8 transcripts in in vitro cultured granulosa cells stimulated with BMP-4 (50 ng/ml) or BMP-7 (100 ng/ml) for 72 h. All values are shown as mean ± SEM. Different superscripts denote statistically different values (*p* < 0.05). Abbreviations: WT indicate wild type cells, WT + BMP-4/BMP-7 indicate wild cells treated with BMP-4 (50 ng/mL), KO indicate BMPR-IB gene knock out cells. KI + BMP-4/BMP-7 indicate KI cells treated with BMP-4(50 ng/ml) or BMP-7 (100 ng/ml).

On expression analysis, Smad-1 revealed a significant (*p* < 0.05; Fig. 6A,B) up regulation in both BMP-4 and BMP-7 stimulated knock in and wild cells in comparison to untreated wild or knock-in cells. Smad-5 transcripts were abundant in both knock in and wild cells co-cultured with BMP ligands, highest being in the former (*p* < 0.05; Fig. 6C,D). Smad 8 transcripts were on average 1.5 and 2 folds higher in wild and knock-in cells treated with BMP-4 and BMP-7 (*p* < 0.05; Fig. 6E,F).

##### Effects on granulosa cell steroidogenic pathway and gonadotropin sensitivity

**Figure 7:**
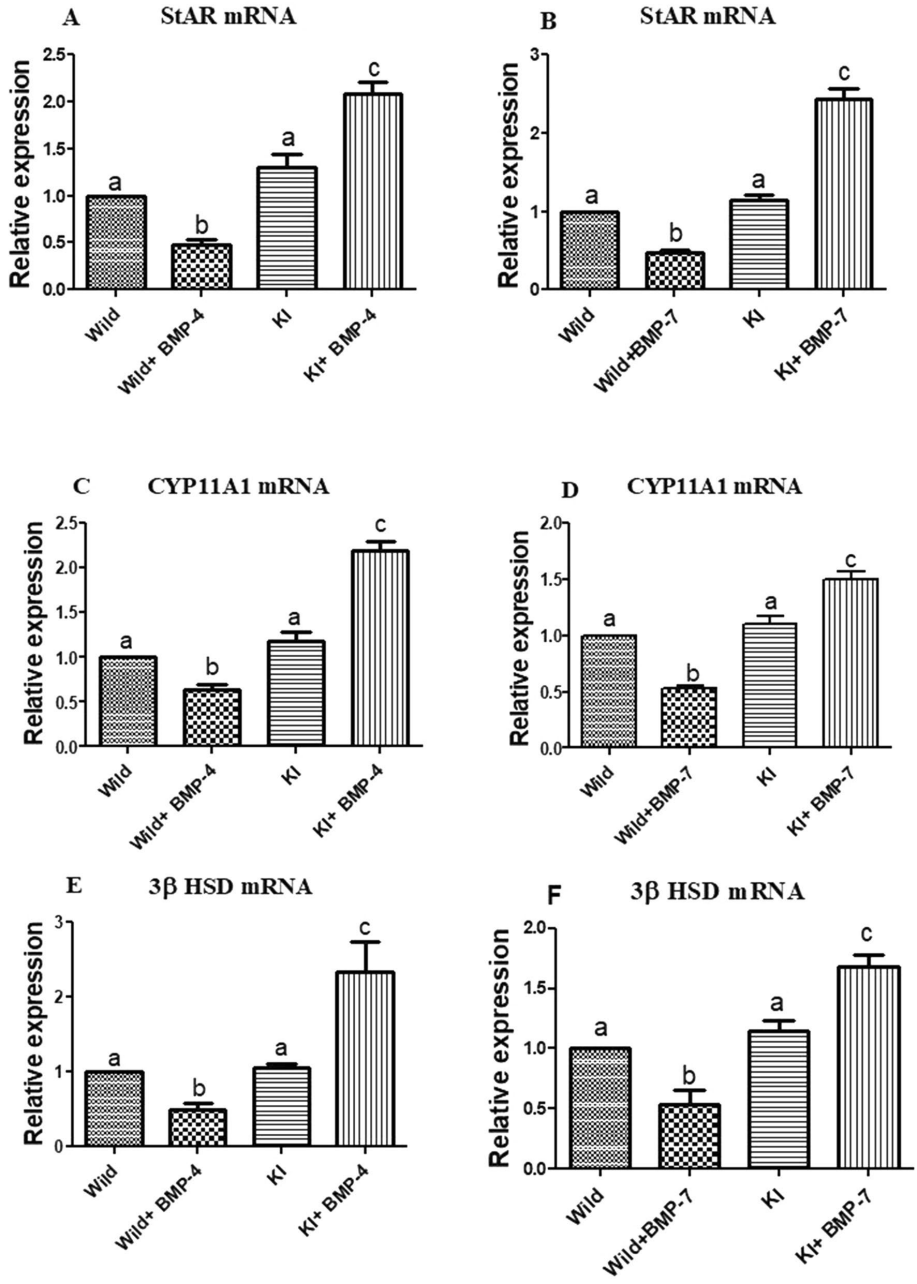
Effect of BMPR-IB gene KI on expression of StAR, CYP11A1 and 3βHSD genes in in vitro cultured granulosa cells stimulated with BMP-4 (50 ng/ml) or BMP-7 (100 ng/ml) for 72 h. All values are shown as mean ± SEM. Different superscripts denote statistically different values (*p* < 0.05). Abbreviations: WT indicate wild type cells, WT + BMP-4/BMP-7 indicate wild cells treated with BMP-4 (50 ng/mL), KI indicate BMPR-IB gene knock out cells. KI + BMP-4/BMP-7 indicate KI cells treated with BMP-4(50 ng/ml) or BMP-7 (100 ng/ml).

**Figure 8:**
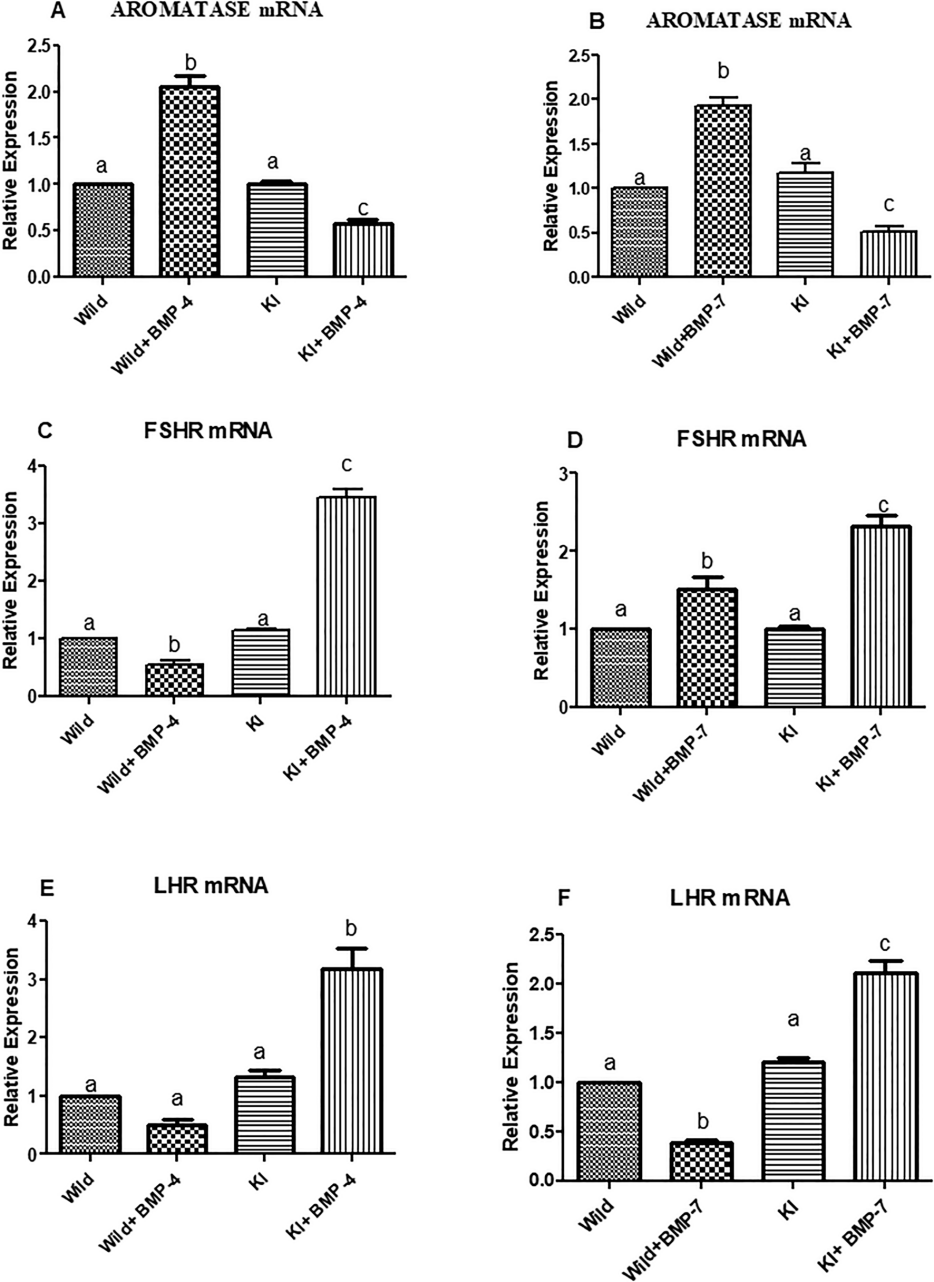
Effect of BMPR-IB gene KI on expression of Aromatase, FSHR and LHR genes in in vitro cultured granulosa cells stimulated with BMP-4 (50 ng/ml) or BMP-7 (100 ng/ml) for 72 h. All values are shown as mean ± SEM. Different superscripts denote statistically different values (*p* < 0.05). Abbreviations: WT indicate wild type cells, WT + BMP-4/BMP-7 indicate wild cells treated with BMP-4 (50 ng/mL), KI indicate BMPR-IB gene knock out cells. KI + BMP-4/BMP-7 indicate KI cells treated with BMP-4(50 ng/ml) or BMP-7 (100 ng/ml).

Wild cells treated with either of the BMP ligands have shown a significant decrease in the expression StAR transcripts. On the other hand, the expression of the same is twofold higher in knock out cells stimulated with BMP-4/7 (*p* < 0.05; Fig. 7A,B). The P450scc (CYP11A1) too has followed the same trend as that of StAR with a highest and lowest expression being in BMP treated knock in cells and wild cells respectively (*p* < 0.05; Fig. 7C,D). The 3βHSD mRNA expression analysis revealed a significant up regulation in knock in cells co-cultured with a BMP ligand (BMP4/7) in comparison to untreated knock in or wild cells (*p* < 0.05; Fig. 7E,F). However, a significant reduction was seen in wild cells cultured with a BMP ligand. Aromatase was significantly expressed to a higher extent in BMP-4/7 treated wild cells. Wherein, its expression was least in BMP ligand stimulated granulosa cells having the FecB mutation and is around 0.5 fold less than that of untreated knock in or wild cells (*p* < 0.05; Fig. 8A,B). Overall, the expression analysis of the above genes in knock-in cells treated with BMP-4/7 has followed a similar pattern when compared with BMPR-IB KO cells treated with BMP-4. A significant decrease and increase in the FSHR transcripts was observed in WT cells cultured with BMP-4 and BMP-7 respectively. Whereas, the FSHR transcripts were significantly upregulated in KI cells stimulated with BMP-4/7 (*p* < 0.05; Fig. 8C,D). A significant downregulation of LHR gene in WT cells stimulated with either of the BMPs was observed along with a significant down regulation (*p* < 0.05; Fig. 8E,F).

##### Effects on cell proliferation and viability

**Figure 9:**
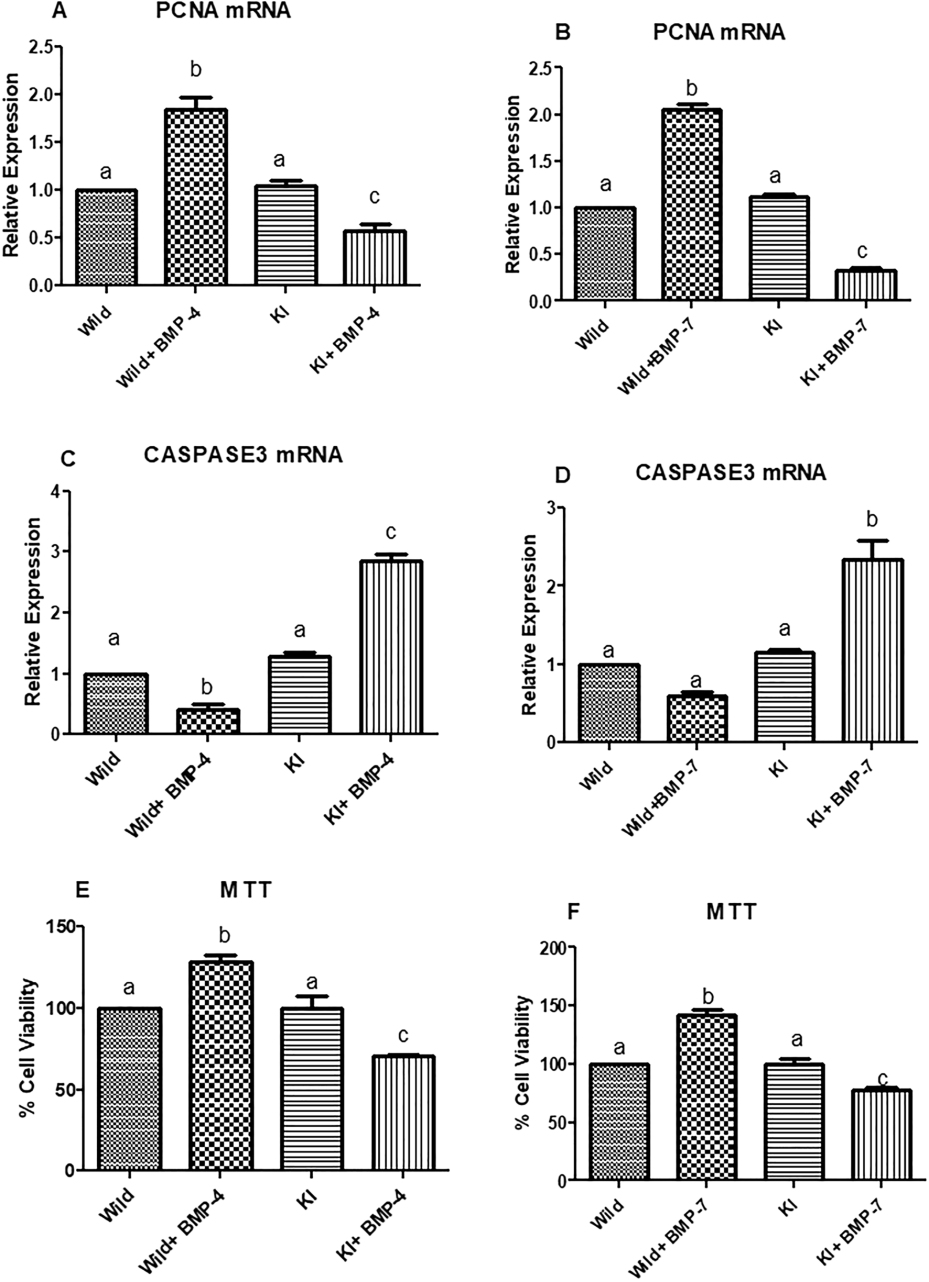
Effect of BMPR-IB gene KI on expression pattern of PCNA, Capase3 genes and cell viability of in vitro cultured granulosa cells stimulated with BMP-4 (50 ng/ml) or BMP-7 (100 ng/ml) for 72 h. All values are shown as mean ± SEM. Different superscripts denote statistically different values (*p* < 0.05). Abbreviations: WT indicate wild type cells, WT + BMP-4/BMP-7 indicate wild cells treated with BMP-4 (50 ng/mL), KI indicate BMPR-IB gene knock out cells. KI + BMP-4/BMP-7 indicate KI cells treated with BMP-4(50 ng/ml) or BMP-7 (100 ng/ml).

A significant higher abundance of Caspase3 mRNA with a concurrent down regulation of PCNA (*p* < 0.05; Fig. 9A,B) was found in BMP treated KI cells. On the other side, Caspase 3 was down regulated (*p* < 0.05; Fig. 9C,D) and PCNA was up regulated in WT cells that have been supplemented with BMP-4 or BMP-7. MTT assay revealed a significant reduction of cell viability of KI cells with BMP stimulation. The cell viability of untreated knock in cells is similar to that of wild cells without BMP treatment. On the other side, there is a significant increase in the viable cell number in wild cells (*p* < 0.05; Fig. 9E,F) that have been supplemented with BMP-4/7 viz almost 130% in comparison to untreated wild or knock-in cells.

##### Effect on estradiol (E2) and progesterone (P4) production

**Figure 10:**
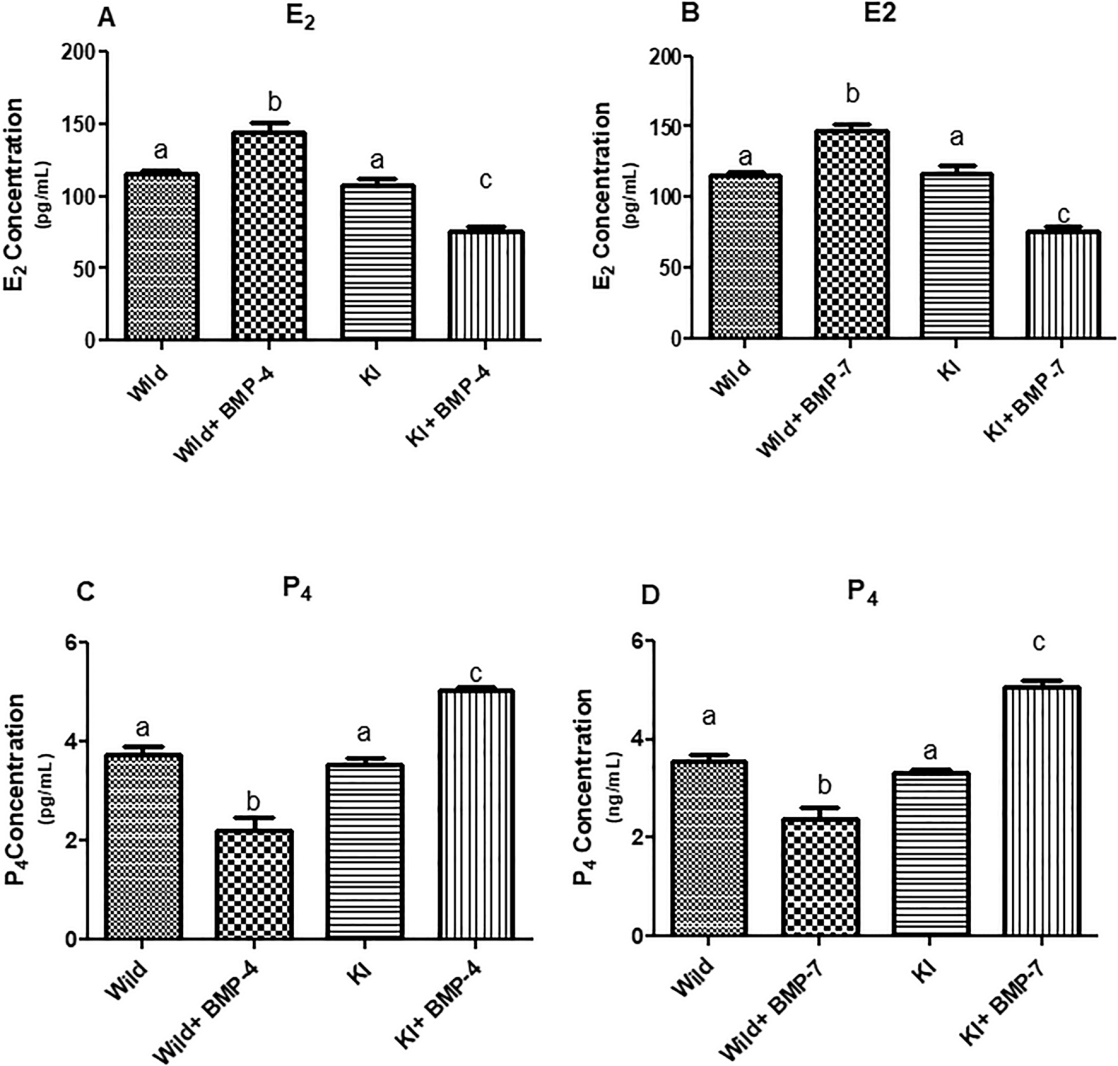
Effect of BMPR-IB gene KI on P4 and E2 synthesis in in vitro cultured granulosa cells stimulated with BMP-4 (50 ng/ml) or BMP-7 (100 ng/ml) for 72 h. All values are shown as mean ± SEM. Different superscripts denote statistically different values (*p* < 0.05). Abbreviations: WT indicate wild type cells, WT + BMP-4/BMP-7 indicate wild cells treated with BMP-4 (50 ng/mL), KI indicate BMPR-IB gene knock out cells. KI + BMP-4/BMP-7 indicate KI cells treated with BMP-4(50 ng/ml) or BMP-7 (100 ng/ml).

There was a significant increase in the estradiol and decrease in the progesterone concentrations in spent media of WT cells cultured with BMP-4/7. Knock-in cells stimulated with BMP-4 or BMP-7 has significantly altered levels of E2 (*p* < 0.05; Fig. 10A,B) and P4 (*p* < 0.05; Fig. 10C,D) production in comparison to BMP treated wild cells or untreated knock in cells.

## Discussion

The complex regulation of follicular dynamics dictates the degree of prolificacy in different species, thus accreting adequate knowledge in this area is necessary to augment livestock production. Follicular development involves rigid intraovarian control mechanisms in conjunction with systemic signals culminating in a series of steps mainly the recruitment, selection and growth of follicles from the primordial stage through to ovulation and corpus luteum formation^33^. Oocyte-somatic cell interactions are now widely accepted to be critical for the development of a follicle as a whole and critical for the prevention of luteinization by promoting growth and regulating steroidogenesis. With a fully established BMP system in the mammalian ovary, mutations in the BMPR-IB, BMP15 and GDF9 genes that have additive effect on ovulation rate have been discovered in various sheep breeds across the world^34^. However, none of these mutations were found in goats^35^. Hence, in our current study, we attempted to document the expression of BMP receptors and the effects of modulating BMPR-IB gene on granulosa cell function in goats.

In our study, we have characterised the expression of BMP receptors (BMPRs) in granulosa cells from antral follicles of various diameters in goat. We found that all the BMP receptors viz. BMPR-IA, BMPR-IB and BMPR-II were expressed in granulosa cells derived from antral follicles of different sizes in regulated manner with stage specific differences. Our result was consistent with the findings of Silva et al.^36^, reported the expression of BMPRs in granulosa cells of antral follicles in goat. Significantly (*p* < 0.05) higher abundance of BMPR-IB mRNA in granulosa cells obtained from large antral follicles was found in our study is in accordance to earlier reports in goat, pig and buffalo^37–39^. The expression of BMPRs in the follicle suggests the potential role of BMP system in affecting the steroidogenesis and functional differentiation of granulosa cells.

Bone morphogenetic proteins (BMPs), belonging to TGF-β superfamily play multiple roles in regulating cellular growth, differentiation and apoptosis in a wide variety of tissues. BMP-4 and BMP-7 are well-documented paracrine/autocrine modulators of granulosa cell steroidogenesis, peptide secretion and proliferation^39,40^. Hence, we have chosen BMP-4 and BMP-7 as ligands in our in vitro study to investigate the outcomes of BMPR-IB gene knock out or knock-in on granulosa cell function.

To determine the effects of BMPR-IB disruption on granulosa cell function, we have successfully knocked out BMPR-IB gene using CRIPSR-Cas genome editing technology and stimulated with BMP-4. Our results on R-Smad expression surprisingly showed a higher transcriptional abundance in knock out cells treated with the BMP ligand in comparison to BMP-4 stimulated control group over the 72 h period. In an earlier study, the deletion of BMPR-IB resulted in an increased level of p-SMAD1/5/9 up on BMP stimulation and have an equally efficient nuclear translocation in pre-osteoblasts in mice^41^. Furthermore, our study revealed a decreasing trend in the expression of StAR, CYP11A, and 3βHSD mRNA in WT cells stimulated with BMP-4, being reversed in knock out cells with BMP-4 treatment. These results are in harmony with the findings that BMP-4 inhibits progesterone secretion by inhibiting StAR and CYP11A1 (P450scc) in granulosa cells of ovine^42^. On the other hand, we found a significant increase in the aromatase expression in BMP-4 treated WT cells, which was abolished by knocking out of BMPR-IB gene. Yi et al.^43^, reported a reduction in levels of aromatase (CYP19A1) production in granulosa cells of mice deficient with BMPR-IB gene. In addition, increased levels progesterone (P4) and lower estradiol (E2) levels in the spent media of knock out cells stimulated with BMP-4 are in agreement with the expression pattern of StAR, CYP11A1, 3βHSD and aromatase transcripts. Altered levels of P4 and E2 suggests that BMP-4 failed to stimulate granulosa cells due to the disruption in the BMPR-IB gene. A significant increase in expression pattern of FSHR and LHR in BMP-4 stimulated KO cells was found suggesting an altered gonadotropin sensitivity. Earlier reports suggest that BMP4 via BMPR-IB downregulates the expression StAR, CYP11A1 and FSHR genes in the granulosa cells which in turn will lead to an inhibition on progesterone synthesis and the early onset of the LH surge and ovulation^44^. With regard to cell viability, our study revealed a significant reduction in the number of viable cells in BMP-4 treated KO cells, where as there is a significant increase in the viable cell count in WT cells cultured with BMP-4. The result regarding the cell viability is in congruence with the expression pattern on pro-apoptotic Caspase3 and PCNA. Earlier report in bovine antral follicles suggest an increased cell viability in granulosa cells cultured with BMPs^40^. In another study, siRNA mediated repression of the BMPR-IB gene in porcine granulosa cells had resulted in a significant inhibition on proliferation and estradiol production, whilst inducing apoptosis^38^. As a whole, knocking out of BMPR-IB gene might have relieved the inhibitory action of BMP-4 on progesterone secretion, with a concomitant decrease in the E2, cell viability and an increased gonadotropin receptivity.

In order to validate the effects of FecB mutation in granulosa cells of goats, we have also introduced the Booroola (FecB) mutation using Easi-CRISPR technology. Then, the BMPR-IB gene knock-in granulosa cells were cultured separately with BMP-4 and BMP-7. In this current study, investigation on the expression pattern of R-Smads transcripts in knock-in cells up on stimulation with both the BMP ligands revealed an upregulation which is similar to the BMP-4 stimulated knock out cells. This suggests that the introduction of Booroola mutation has resulted in a loss of function of BMPR-IB. Earlier studies reported that FecB mutation attenuates the receptor activity partially or completely leading to an altered signaling pathway resulting in high ovulation rate in sheep^45,46^. Additionally, the upregulation of StAR, P450scc and 3βHSD genes along with a significant downregulation of aromatase in the BMP-4/7 treated knock in cells is analogous to the pattern that has been found in BMP-4 treated knock out cells. In our study, high P4 concentration in spent media in either of the BMP stimulated knock-in cells is in parallel with the expression of StAR,P450scc, 3βHSD genes and P4 levels in BMPR-IB KO cells. Earlier reports in granulosa cells from FecB carrier ewes revealed a higher P4 secretion than those from non-carrier animals, suggesting FecB mutation has resulted in loss of responsiveness to the inhibitory effect of BMP-4^30^. The lower E2 levels in knock-in cells treated with BMP-4/7 found in our current investigation was comparable to that of the findings in KO cells. McNatty et al.^14^, found that oestradiol from the five or more preovulatory follicles from FecB carrier ewes is compared to one or two such follicles of non-carriers. A decrease in oestradiol and inhibin production was observed in follicles from FecB carriers^13^. In addition, our investigation to test gonadotropin sensitivity revealed an upregulation in both FSHR and LHR transcripts in granulosa cells with FecB mutation. Previous studies report that FecB mutation results in the precocious maturation of ovarian follicles by increasing the sensitivity of the follicles to FSH, developing LHR and aromatase activity earlier and ovulate smaller follicular diameters^46–48^. Moreover, a significant reduction in viability of the BMP treated knock-in cells was revealed in MTT assay which is in accordance with down regulated PCNA and up regulated Caspase3 transcripts. This could be due to the probable loss of receptor function in knock-in cells leading to a failure in inducing proliferative and anti-apoptotic effects of BMPs resulting in ovulatory follicles with smaller diameters. Another study with luciferase reporter, granulosa cells with FecB mutation showed no response suggesting a loss of function in the receptor activity^49^.

## Conclusion

The study has revealed an altered Smad signaling, steroidogenesis and cell viability upon modulation of BMPR-IB gene in granulosa cells similar to that are documented in sheep breeds carrying the FecB mutation. These findings suggest the probable effects of BMPR-IB gene modulation on granulosa cell function of goats can potentially alter their reproductive performance.
